# A Survey of German Dentists on the Prophylactic Use of Antibacterial Drugs in Patients in Risk Groups: Diabetes, Joint Replacement, Risk of Endocarditis, and Immunosuppression or Organ Transplantation

**DOI:** 10.1155/ijod/8452465

**Published:** 2026-06-19

**Authors:** Joshua Kinzel, Roland Seifert

**Affiliations:** ^1^ Institute of Pharmacology, Hannover Medical School, Hannover, Germany, mh-hannover.de

**Keywords:** antibacterial drugs, antibacterial prophylaxis, dentistry, dentists, guidelines, survey

## Abstract

**Introduction:**

Antibacterial drugs (ABDs) prescribed by dentists account for a significant proportion of all prescriptions for such drugs in Germany. The number of prescriptions is by no means uncontroversial, as guidelines are often lacking and antibacterial stewardship (antibiotic stewardship) calls for greater caution in the use of ABD. A survey was therefore conducted to answer the question of under what indications and with what consistency dentists in Germany use ABD.

**Methodology:**

In the survey, German dentists were asked to indicate the frequency of their ABD prescriptions for various dental procedures or comorbidities in four patient groups: patients at risk of endocarditis, joint replacement, diabetes, and organ transplantation or immunosuppression. They were also asked about the type of primary and secondary medication used. The responses were then summarized to form an overall picture and briefly compared with German‐language guidelines. In addition, the year of state examination and the perceived level of confidence in using ABD were determined.

**Results:**

It was found that only in patients with a risk of endocarditis and organ transplantation or immunosuppression did some questions show a positive trend, and in patients with diabetes and joint replacement, a negative trend up to a negative consensus. In all cases, the primary ABD prescribed was amoxicillin and the secondary was clindamycin by an overwhelming majority of responses.

**Discussion:**

The inconsistent results indicate a great need for education on the use of prophylactically prescribed ABD. Particularly in cases of rare pre‐existing conditions, there is a high level of uncertainty with a tendency toward prophylactic drug administration. Existing transnational guidelines ensure a more uniform approach overall.

**Conclusion:**

Although no widely consistent approach could be identified for the various questions, the use of ABD appears to be generally restrained.

## 1. Introduction

Antibacterial drugs (ABDs) have been used frequently in human medicine for many years and are also an indispensable part of the range of medications prescribed in dentistry. In terms of absolute prescription figures, ABDs rank second behind fluoride preparations [1]. Overall, prescriptions by dentists accounted for just under 9% of all ABDs prescribed [2]. As with any medication, the desired positive effects of reducing pathological bacteria must be weighed against the potential negative effects. Of particular note here is the development of resistance, which has received increasing attention in recent years in the context of antibacterial stewardship. In order to enable doctors and dentists to weigh up the risks and benefits of prescribing ABDs on a scientifically sound basis, professional associations are in many cases developing guidelines and recommendations for action based on studies. However, it has been found that in dentistry, the guidelines of professional associations regarding the indication, dose, and duration of medication administration are often inconsistent and leave dentists with considerable leeway [3, 4]. The only area where there is broad consensus is endocarditis prophylaxis, since the American Heart Association (AHA) published guidelines in 2007 recommending endocarditis prophylaxis only for high‐risk patients, thereby significantly reducing the target group compared to previous guidelines. These guidelines have been adopted almost in their entirety by a large number of professional associations across countries and even today have a major influence on the recommendations for ABD in other pre‐existing conditions [5–7]. Surveys have already shown that dentists in the United States of America follow these guidelines. However, the survey also showed that the recommendations of the American Academy of Orthopaedic Surgeons for patients with prosthetic joint replacements did not result in this uniform approach to endocarditis prophylaxis [8]. In Germany, there is now only a broad consensus on the recommendations for administering ABD during dental implantation in patients with diabetes, immunosuppression, antiresorptive therapy, or a risk of endocarditis [9–12]. This is true even though the recommendations themselves emphasize that the medication can only increase the success of the implantation and does not prevent systemic infection. The situation is very similar across Europe. A study from 2024 found that some European experts recommend prophylaxis with ABD for dental implants, but overall, more ABD is prescribed for prophylaxis than recommended. Therefore, more education is needed to promote a more rational approach [13]. A study conducted in 2019, which surveyed dental students in Italy on prophylaxis with ABD in endodontic procedures, came to the same conclusion [14]. In these surveys, the respondents mostly deviated from the recommendations in that they tended to prescribe ABD more than the recommendations suggested. A further study from Italy, which this time surveyed licensed dentists, a survey of Swiss dentists, and a cross‐sectional survey at a university in Saudi Arabia came to a similar conclusion and found a wide variation in indications [15–17]. There are also critical international voices that generally question the benefits of prophylaxis with ABD in implantology [18]. The fact that there are few and no uniform guidelines for other patient groups is probably due to the fact that although the effectiveness of ABD in preventing bacteremia has been proven in studies [19], the study situation regarding the risk of bacteremia due to dental procedures and its significance is very poor. Although bacteremia can be detected in many patients after dental procedures, this depends heavily on the type of procedure, and various studies have shown very different pictures of the percentage frequency of bacteremia and the duration or detection of bacteria in the blood of the test subjects. In addition, bacteremia has also been detected in some studies during chewing, tooth brushing, flossing, and similar activities [20]. However, permanent prophylaxis with ABD is not carried out for such everyday activities and is not recommended by any professional association. It seems likely that this has an impact on the distribution of prescriptions. In addition, it is possible that not all treating dentists are aware of the existing guidelines and their updates. Both points would have an impact on the medical care situation, as decisions would then have to be made on the basis of anecdotal evidence from one’s own experience or that of colleagues. This would weaken the general goal in medicine of having a uniform, scientifically‐based approach across the board. This raises the question of how consistent dentists are in their everyday practice when administering ABD and in which procedures such drugs are used at all. This will be determined in the following by surveying dentists in Germany about their approach to certain patient groups. The original questionnaire in German, including the response options, can be found in the Supporting Information (questionnaire_base). The visualization of the results can be found under the relevant subheading in the results section, with the corresponding reference.

## 2. Methodology and Materials

In the survey, which was only available online, dentists working in Germany were asked about their prescribing behavior with regard to prophylactic ABD. The survey focused exclusively on systemic ABD and was divided into four main areas: ABD for patients with endocarditis, patients with organ transplants or immunosuppression, diabetes, and joint replacements. In each area, there were questions that addressed different forms and manifestations of the disease, as well as questions related to various dental activities. For each question, the response options were never (0%), rarely (<25%), occasionally (25%–50%), frequently (50%–75%), very frequently (>75%), always (100%), and “not specified,” whereby the estimated percentage of patients in each group who received prophylactic treatment with ABD from the survey participant should be indicated. In addition, for each main area, the question was asked about the most frequently and second most frequently used prophylactic ABD. The possible answers were limited to amoxicillin, clindamycin, penicillin V, doxycycline, metronidazole, ampicillin, vancomycin, azithromycin, clarithromycin, and “not specified.” A “yes”/“no” option was used to ask whether the participating person felt confident overall in using ABD for prophylactic purposes. Finally, the year of the state examination was to be indicated, with a selection ranging from 1950 to 2023. Each question had to be answered. Only fully completed surveys were included in the evaluated results, resulting in 537 evaluable questionnaires from 1070 participants. Participation in the survey was voluntary, and the link to the survey was distributed to members via the dental associations of Saarland, Berlin, Baden‐Württemberg, Lower Saxony, Mecklenburg‐Western Pomerania, Westphalia‐Lippe, and Thuringia. The survey was available online on the SoSci internet platform for a period of 5 months from 10.2023 to 03.2024. After the deadline, the results were transferred to Excel spreadsheets by the SoSci and evaluated by the author of the study. No further checks were carried out. It was not possible for German dentists to participate or comment retrospectively.

At the beginning of the survey, potential participants were shown the following text: “The purpose of this survey is to statistically record the use of prophylactically prescribed antibiotics in everyday practice. The survey takes ~5–10 min to complete. By participating, you are making an important contribution to my research. I would therefore be very grateful if you would take part in my survey. Participation is voluntary. All information you provide is anonymous and will not be evaluated on an individual basis.“ In order to proceed to the first question, participants had to tick the box “I agree to participate.”

The responses were first evaluated graphically in the form of pie charts and bar charts. To assess the data obtained more accurately, each question was then classified according to the different degrees of agreement. If more than 80% of respondents answered “always” and “very often” or “never” and “rarely” to a question, this was evaluated as a clear positive or negative consensus on the use of ABD in the specific case. If between 50% and 80% of participants answered “always” and “very often” or “never” and “rarely,” this was considered a positive or negative trend. All other results were interpreted as inconclusive. Finally, the results were briefly compared with the recommendations of German professional associations.

## 3. Results

### 3.1. Prophylactic ABD for Endocarditis Prophylaxis

As shown in Table 1, there is a clear consensus regarding the administration of ABD only in cases of prosthetic valve replacement. In patients who had survived bacterial endocarditis and heart transplant patients, a positive trend toward endocarditis prophylaxis was still evident. In cases of cyanotic heart defects, congenital cyanotic heart disease, hypertrophic cardiomyopathy, mitral valve prolapse, acquired valve dysfunction, and pulmonary shunts, the data indicate an inconclusive approach. No majority opinion in favor of or against the use of ABD for prophylactic purposes in any of the aforementioned heart diseases in combination with additional risk factors such as smoking or heavy alcohol consumption could be found. The survey on the type of procedure indicating endocarditis prophylaxis did not reveal a clear positive consensus. Only a positive trend was observed for tooth extraction, implantation, periodontal procedures, and procedures involving the bone. A rather negative trend was observed for inflammation of the oral mucosa, injury to the mucosa, injury to the gingiva, local anesthetic injection, and probing of the periodontal pocket. A consensus could only be reached on not administering ABD in cases of postoperative suture removal. A mixed picture emerged when it came to the question of whether ABD should be used for endocarditis prophylaxis in cases of endodontic inflammation and dental and maxillofacial trauma. A comparison of these results with the guidelines of the German Society of Dentistry and Oral Medicine (DGZMK) and the German Society for Cardiology and Cardiovascular Research (DGK) (see Table 2) shows that the recommendations are largely implemented. Significant deviations can only be found in endocarditis prophylaxis for procedures involving injury/manipulation of the gingiva and local anesthetic injections.

**Table 1:** Use of ABD for endocarditis prophylaxis expressed as a percentage, self‐created image.

Use of ABD for endocarditis prophylaxis	Always	Very often	Frequently	Occasionally	Rarely	Never	No information
Cyanotic heart defect	36	9	7	7	12	12	17
Prosthetic heart valve	72	10	7	4	4	1	2
Survived bacterial endocarditis	68	10	6	4	4	3	5
Heart transplant patients	69	5	3	2	2	1	18
Cyanotic congenital heart disease	30	10	6	9	9	10	26
Surgically implanted pulmonary shunts	19	10	7	12	11	13	28
Other congenital heart defects (except cyanotic)	13	7	7	15	20	16	22
Hypertrophic cardiomyopathy	7	3	5	7	15	32	31
Mitral valve prolapse	18	6	6	8	17	23	22
Acquired valvular dysfunction (e.g., rheumatic heart disease)	15	5	8	8	13	18	33
With a heart condition not listed in the survey	5	3	4	9	13	21	45
With one of the above conditions in combination with other risk factors	22	8	10	11	11	14	24
For tooth extraction	51	9	6	9	14	7	4
At implantation	46	6	6	4	6	6	26
In cases of endodontic inflammation	22	9	10	14	21	19	5
For inflammation of the oral mucosa	6	4	5	11	25	43	6
In periodontal procedures	43	8	9	12	16	8	4
In cases of dental or jaw trauma	30	6	8	12	18	14	12
With mucosal damage	10	4	6	8	21	42	9
In cases of gingival injury	11	3	5	7	20	47	7
With local anesthetic injection	12	2	4	3	13	62	4
When probing the PA gap	16	4	6	5	17	47	5
With postoperative suture removal	5	2	1	3	9	76	4
For procedures involving bone	48	10	7	9	11	7	8

**Table 2:** AHA recommendations for endocarditis prophylaxis based on Naber et al. [12].

Indications for endocarditis prophylaxis	Dental procedures for which endocarditis prophylaxis is recommended
Heart valve replacement (mechanical and biological valves)	Tooth extraction/osteotomy/root tip resection/implantation
Patients with reconstructed valves using alloprosthetic material in the first 6 months after surgery	Professional teeth cleaning/scaling
Expired endocarditis	Biopsies/mucosal interventions/resections
Untreated (or palliatively treated with systemic‐pulmonary shunt) cyanotic heart defects	Intraligamentary anesthesia, injection into infected area
Surgically treated heart defects with implantation of conduits (with or without valve) or residual defects (meaning turbulent blood flow) in the area of the prosthetic material	Measures involving manipulation of the gingiva: – Placement and removal of orthodontic bands – Retraction cords – Separation of teeth
Successfully operated heart defects, provided that prosthetic material has been inserted (over 6 months)	Endodontic treatments
Patients after heart transplantation with impaired valve function	—

**Figure 1:**
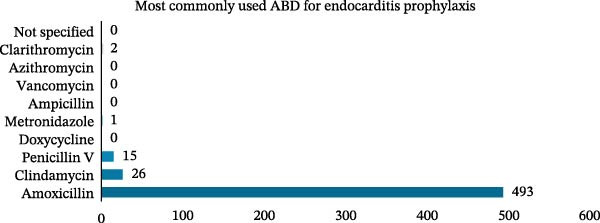
Most frequently used ABD for endocarditis prophylaxis in absolute numbers.

**Figure 2:**
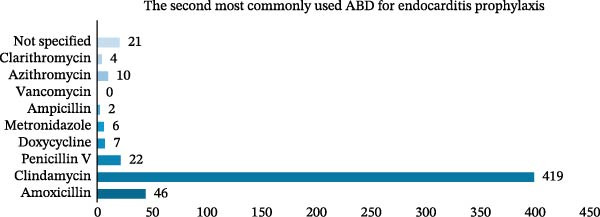
Second most commonly used ABD for endocarditis prophylaxis in absolute numbers.

The questions about the most frequently and second most frequently used ABD yielded a clear result, with amoxicillin being the most frequently used drug and clindamycin the second most frequently used (see Figures 1 and 2). This is consistent with the recommendations of the guidelines, which also prefer amoxicillin for the basic case and recommend clindamycin only as a second‐line ABD in cases of penicillin allergy, for example [12].

### 3.2. ABD Used Prophylactically in Patients With Joint Replacements

The survey revealed the following picture, shown in Table 3, regarding the prescription of ABDs in patients with joint replacements. It is striking that no positive consensus or positive trend could be identified for any of the questions, which suggests a generally critical approach to prophylaxis with ABDs in this patient group. A negative trend was observed in dental procedures following joint surgery more than 2 years ago, in hip replacement, in patients with joint replacement and other risk factors, as well as in tooth extraction, endodontic inflammation, periodontal procedures, and dental and maxillofacial trauma. In cases of inflammation of the oral mucosa, injury to the mucosa or gingiva, local anesthetic injection, postoperative suture removal, and probing of the PA gap, the dentists participating in the survey agreed that there was a consensus against the use of ABD. A wide variety of approaches among dental practitioners and thus an unclear picture emerged in response to questions about dental procedures within 2 years of joint surgery, implantation, and procedures involving the bone. No trend could be identified from the responses. In its recommendations, the German Society for Endoprosthetics recommends the administration of ABD for invasive dental procedures [21]. Thus, there is a clear discrepancy between the approach taken by dentists and the recommended procedure in cases of tooth extraction, periodontal procedures, and dental and maxillofacial trauma. Even in cases of implantation and procedures involving the bone, the recommended procedure is obviously not applied in every treatment.

**Table 3:** Use of ABD in patients with joint replacement expressed as a percentage, self‐created image.

Use of ABD for patients with joint replacements	Always	Very often	Frequently	Occasionally	Rarely	Never	No information
During dental procedures within 2 years after joint replacement surgery	23	10	8	14	23	20	2
More than 2 years after joint replacement surgery	6	4	5	9	24	50	2
For hip replacement	14	10	7	11	22	32	4
In patients with joint replacement in combination with other risk factors	13	9	7	13	20	32	6
For tooth extraction	15	7	6	15	32	23	2
At implantation	23	6	7	8	15	13	28
In cases of endodontic inflammation	6	6	9	22	27	28	2
For inflammation of the oral mucosa	1	1	3	9	28	56	2
In periodontal procedures	12	7	7	18	35	18	3
In cases of dental or jaw trauma	9	4	6	15	27	25	14
In case of mucosal injury	2	2	3	6	19	63	5
In cases of gingival injury	2	2	2	3	21	67	3
With local anesthetic injection	3	1	1	2	10	81	2
When probing the PA gap	3	1	2	4	16	72	2
With postoperative suture removal	2	0	1	1	6	88	2
For procedures involving bone	17	8	8	18	29	12	8

**Figure 3:**
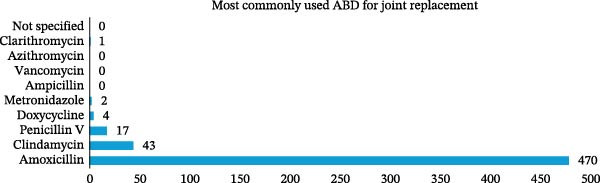
Most commonly used ABD in joint replacement in absolute numbers.

**Figure 4:**
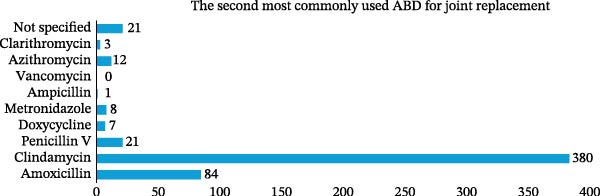
Second most commonly used ABD for joint replacement in absolute numbers.

Even in patients with joint replacements, the vast majority of dentists rely on standard ABD in dentistry. Amoxicillin is also the most commonly used drug here, with clindamycin usually used as a reserve in cases of contraindications to amoxicillin (see Figures 3 and 4). This is in line with the recommendation of the German Society for Endoprosthetics, which recommends a single 2 g dose of amoxicillin preoperatively [21].

### 3.3. Prophylactic ABD in Patients With Diabetes

The results of the survey on the administration of prophylactic ABD in patients with diabetes show a mixed picture, which is generally critical of prophylaxis. As can be seen in the survey of patients with joint replacements and Table 4, there was no question that achieved a positive consensus or a positive trend. The survey revealed a consensus against the use of ABD in both type 1 and type 2 diabetes mellitus. In addition, most dentists do not use ABD prophylaxis in cases of inflammation of the oral mucosa, injury to the mucosa or gingiva, local anesthetic injection, probing of the PA gap, and postoperative suture removal. There is somewhat less agreement and therefore only a negative trend in patients with poorly controlled diabetes, diabetes in combination with other risk factors, tooth extraction, endodontic inflammation, periodontal surgery, and dental and maxillofacial trauma. The picture is unclear for dental procedures involving tooth extraction and implantation, as well as for procedures involving bone. In the field of diabetes, there is only one guideline for the use of ABD in implantation in German‐speaking countries, issued by the German Society of Dentistry and Oral Medicine in conjunction with the German Diabetes Society [9]. This guideline recommends the use of ABD. The ambiguous approach in the survey therefore deviates from the guidelines.

**Table 4:** Use of ABD in patients with diabetes expressed as a percentage, self‐created image.

Use of ABD for patients with diabetes	Always	Very often	Frequently	Occasionally	Rarely	Never	No information
In type 1 diabetes mellitus	1	2	3	10	25	58	1
In type 2 diabetes mellitus	1	2	3	10	26	57	1
With poorly controlled diabetes	5	7	11	15	22	38	2
In diabetes in combination with other risk factors	3	8	9	14	24	40	2
With diabetes with tooth extraction	5	5	7	15	30	36	2
In diabetes at implantation	23	6	6	8	9	24	24
In diabetes in endodontic inflammation	1	4	5	15	27	46	2
In cases of diabetes In cases of inflammation of the oral mucosa	1	1	2	6	24	63	3
With diabetes during periodontal procedures	4	6	6	15	28	38	3
With diabetes with dental or jaw trauma	5	6	6	14	24	37	8
In diabetes in cases of mucosal damage	0	1	1	6	20	67	5
In diabetes in cases of gum injury	0	1	1	4	19	71	4
In diabetes with local anesthetic injection	1	0	0	2	10	84	3
In diabetes when probing the PA space	1	1	0	3	13	79	3
In diabetes In postoperative suture removal	0	0	0	0	7	90	3
In diabetes In procedures involving bone	11	8	10	17	18	29	7

**Figure 5:**
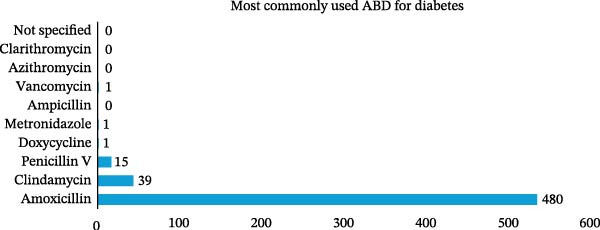
Most commonly used ABD for diabetes in absolute terms.

**Figure 6:**
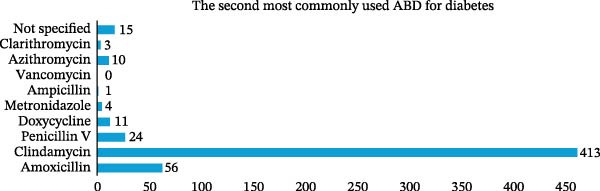
The second most commonly used ABD for diabetes in absolute terms.

In the patient group with diabetes, amoxicillin was the first‐choice drug for prophylaxis, with ABD as an alternative, and clindamycin was the second most commonly used drug in the dental profession (see Figures 5 and 6). The DGZMK and DGI guidelines do not specify which drug should be administered.

### 3.4. Prophylactic Use of ABD in Patients With Immunosuppression or Organ Transplants

According to the survey results presented in Table 5, there is no uniform approach among dentists regarding the prophylactic administration of ABD in immunosuppressed patients or patients who have received an organ transplant. No positive consensus was reached on any of the questions for this patient type either. A positive trend was observed in patients suffering from an immunosuppressive disease, taking immunosuppressive medication, generally after organ transplantation, in patients who were either immunosuppressed or had undergone organ transplantation and had additional risk factors, as well as in dental procedures involving bone, tooth extraction, and implantation. A tendency to reject ABD was observed in cases of inflammation of the oral mucosa, injury to the gingiva or mucosa, local anesthetic injection, probing of the PA gap, and postoperative suture removal. However, no negative consensus was found for any of the questions asked. A mixed picture emerged in cases of endodontic inflammation, periodontal procedures, and dental and maxillofacial trauma. The German Society of Dentistry and Oral Medicine, together with the Working Group for Oral and Maxillofacial Medicine, has published an S2k guideline recommending the use of ABD prior to dentoalveolar procedures [22]. The positive trend toward the use of ABD in dental procedures involving bone, tooth extraction, and implantation is therefore a step in the right direction. However, the guideline is not followed for periodontal procedures and dental and maxillofacial trauma.

**Table 5:** Use of ABD in patients with immunosuppression or organ transplantation expressed as a percentage, self‐created image.

Use of ABD for patients with immunsuppression or organ transplants	Always	Very often	Frequently	Occasionally	Rarely	Never	No information
In patients with immunosuppressive disease	42	14	12	11	6	3	12
In patients taking immunosuppressive medication	37	16	12	13	7	3	12
In patients after organ transplantation	51	12	8	7	5	2	15
In cases of immunosuppression or following organ transplantation in combination with other risk factors	50	12	8	7	3	3	17
For tooth extraction	52	12	8	9	4	3	12
At implantation	46	6	3	5	2	3	35
In cases of endodontic inflammation	24	11	11	12	15	13	14
For inflammation of the oral mucosa	10	4	9	12	22	29	14
In periodontal procedures	36	8	11	12	11	8	14
In cases of dental or jaw trauma	32	7	8	10	11	12	20
In case of mucosal injury	10	4	6	10	21	33	16
In cases of gingival injury	9	3	6	10	22	35	15
With local anesthetic injection	10	2	3	4	15	53	13
When probing the PA gap	11	4	5	7	17	43	13
With postoperative suture removal	6	1	1	2	13	64	13
For procedures involving bone	43	11	10	8	5	5	18

**Figure 7:**
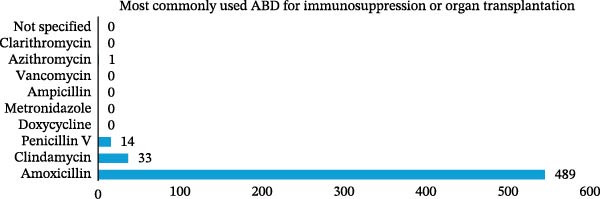
Most commonly used ABD in absolute numbers for immunosuppression or organ transplantation.

**Figure 8:**
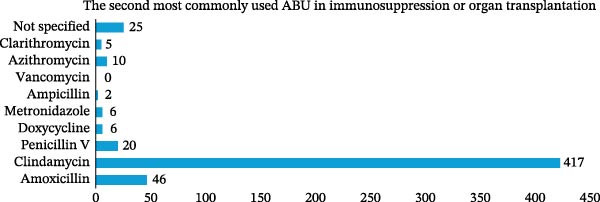
Second most commonly used ABD for immunosuppression or organ transplantation in absolute numbers.

The most commonly used ABDs for prophylaxis were also amoxicillin and, as an alternative, clindamycin in the patient group with immunosuppression or organ transplantation (see Figures 7 and 8). The S2k guideline recommends a single dose of 2 g of amoxicillin and, as a second choice, 0.6 g of clindamycin, which is in line with the results of the survey [22].

**Figure 9:**
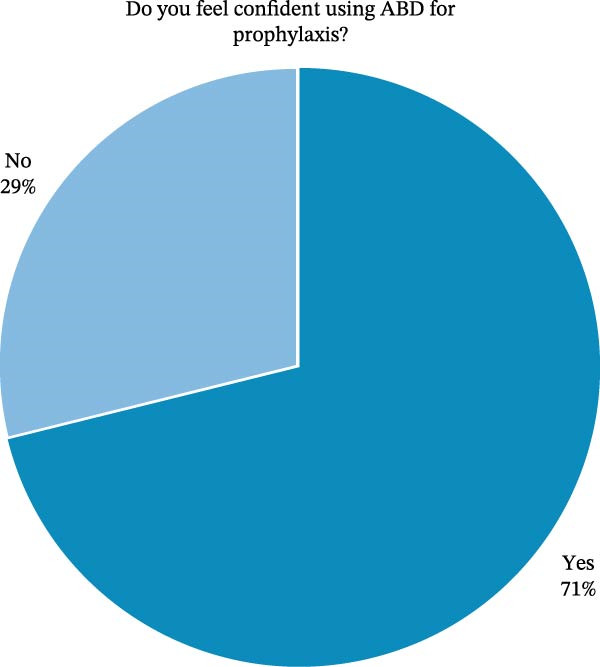
Confidence in using DPI for prophylactic purposes.

**Figure 10:**
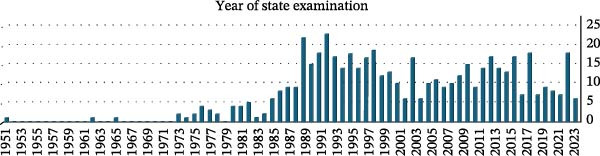
Year of state examination of participants.

Finally, the survey recorded how confident the dentists surveyed felt when using prophylactically prescribed ABD (see Figure 9) and the year in which they passed their state examination (see Figure 10).

### 3.5. Limitation

A total of 537 people completed the survey and answered all questions. According to the German Dental Association, there were 101,344 practicing and nonpracticing dentists in Germany at the beginning of 2023. This means that only slightly more than 0.5% of all dentists in Germany participated in the survey. In addition, the survey was distributed via the dental associations of the federal states, not all of which agreed to participate. The survey was voluntary and neither personalized nor monitored, which does not rule out multiple responses and deliberate misrepresentation. It cannot be ruled out that dentists with a heightened awareness of this issue were more likely to participate in the survey, which may have skewed the results. Neutral questions and anonymity were used in an attempt to influence the dentists’ answers as little as possible. Nevertheless, it cannot be ruled out that some answers reflect social desirability perceived by the participants rather than actual practice. When selecting the ABD used, it was only possible to choose one of the ten options provided or not to provide any information, but it was not possible to provide information outside of this. All of this may have led to a distortion of the survey results. The significance of the results is also limited by the fact that the survey does not record actual prescriptions but only the dentists’ self‐assessment. In terms of the distribution of the period of activity, no exam year was significantly overrepresented, which should ensure that different generations of dentists are well represented.

## 4. Discussion

The results of the survey generally show a rather cautious use of ABD, as well as a wide variation in the type and extent of administration. This is consistent with the surveys of dentists and dental students in Italy, Switzerland, and Saudi Arabia mentioned in the introduction. In these surveys, the authors also found that, even with existing guidelines, there were sometimes significant deviations from them in everyday practice. Amoxicillin was the most commonly used drug in all surveys. When asked about procedures without existing guidelines, the results were similar to the survey of German dentists, with a mixed approach and a tendency to administer AMD. This was interpreted by all authors as a precautionary measure on the part of dentists. In many cases, the dentists’ prescribing behavior was classified as inappropriate or at least unnecessary in the evaluation. The authors unanimously call for the development of binding guidelines. Even though the various surveys have different focuses and are therefore not directly comparable, a few positive points can be highlighted. For example, the surveys showed that German dentists are more aware of the importance of antibiotic stewardship and feel more confident about prescribing antibiotics. There is also a stronger orientation toward the existing international guidelines.

An interesting point of the survey is the division into patient groups that showed a positive trend in the questions. Here, the patient group with endocarditis risk and immunosuppression or organ transplantation is contrasted with the group of patients with joint replacement or diabetes. In contrast to the other two groups, questions with a positive trend were found among patients at risk of endocarditis, immunosuppression, or organ transplantation. The same division can also be found in the distribution of unclear procedures. In addition, the only question that achieved a positive consensus was in the area of endocarditis risk. This can be attributed to the high level of consensus across countries and professional societies on the recommended actions. However, this also means that, despite long‐standing guidelines, there is a lack of consensus on other clinical pictures and procedures. Despite the recommendations of the AHA, DGZMK, and AE, less than 50% of respondents stated that they would use ABD in most cases of cyanotic heart defects, congenital cyanotic heart disease, acquired valvular dysfunction, and pulmonary shunts. In cases of gingival injury, suture removal, and local anesthetic injection, more than half of the respondents even use ABD rarely or never. It is therefore clear that even with endocarditis prophylaxis, with its clearly defined recommendations for action, it has not yet been possible to achieve a largely uniform approach in every area. Nevertheless, a positive effect is evident in direct comparison with the other three areas covered. For the three patient groups, diabetes, joint replacement, and immunosuppression or organ transplantation, the recommendations are much more general. This is reflected in the survey by a greater discrepancy between the recommendation and the actual procedure. In the patient group with organ transplantation or immunosuppression, ABD is given in many cases of procedures that fall under the wording of the recommendation. This area therefore ranks second only to endocarditis prophylaxis in terms of compliance with recommendations for action in everyday dental practice. We explain this by suggesting that practicing dentists weigh up the risk of sepsis or loss of a transplanted organ against a possible allergic reaction to an ABD or the possible development of multiresistant bacteria. The latter usually only causes problems for the patient in the distant future and cannot be linked to the dentist’s behavior in concrete terms. This further increases the pressure to choose the supposedly safer option of administering ABD. Nevertheless, there are no interdisciplinary guidelines in the field of immunosuppression and organ transplantation, nor are there any guidelines for the dental treatment of patients with diabetes and joint replacements [4]. This explains the very mixed picture of dental procedures for patients with these pre‐existing conditions. Most questions with a negative consensus were in the group of patients with diabetes. This is probably due to the fact that dentists come into contact with people with diabetes in their everyday work because of its high prevalence in the population and are generally more familiar with this disease. Continuing education and articles dealing with the disease will do the rest to dispel the fear surrounding it. In addition, many of the questions with a negative consensus involve dental procedures that also received a negative consensus in the other three patient groups. It therefore appears that there are types of dental procedures in which practitioners do not see a high risk of bacteremia in patients. Finally, we would like to explicitly address the question of prophylaxis by ABD during implantation. Although there is no consensus in any group, the number of those who did not provide any information must be taken into account. Since not all dentists place implants, the average number of nonresponses is approximately ¼ of all votes. If these votes were excluded, the result would often show at least a positive trend. This is at least in line with the recommendations and guidelines applicable in Germany and shows a similar result to international surveys [14]. The cautious use of ABD in some patient groups and procedures without guidelines can be taken as an indication that prophylaxis with such drugs is not necessary in such cases. It can be assumed that practicing dentists would have quickly returned to prescribing ABD as soon as a complication could be attributed to it. This should encourage dentists who act differently to reconsider their prescriptions and could help professional associations in developing guidelines.

The German dental profession is unanimous regarding the type of ABD used. With a few exceptions, it appears that the treatment is based on established practice, with amoxicillin prescribed as the primary medication and clindamycin as the secondary medication, regardless of the patient group. Presumably, patients at risk of endocarditis are being transferred to other groups, as, as already mentioned, comprehensive guidelines exist only in this area, which specifically recommend this approach. However, all results must always be viewed in light of the limiting factor that only the responses of 0.5% of all dentists in Germany are represented in this questionnaire. Even though this study attempts to extrapolate the findings to the entire dental profession, in reality, there may be greater variations in the prescription of ABD.

It is interesting to note that when asked whether they feel confident in dealing with ABD, almost three‐quarters of the dentists surveyed answered yes. This result stands in stark contrast to the different prescribing behaviors. One explanation for this is that dentists fall into the trap of anecdotal evidence, combined with the problems mentioned above, which occur late and cannot be directly linked to the administration of medication. Whether drugs were administered unnecessarily is probably given much less consideration than complications occurring as a direct result of dental treatment without drugs. It is therefore all the more encouraging that, taken together, all categories and questions indicate a cautious use of ABD.

## 5. Conclusion and Future Studies

According to the survey, the dental profession is still a long way from the uniform use of ABD throughout Germany. However, a cautious trend is emerging. Ultimately, as called for in most of the articles cited, randomized controlled studies on the prophylactic use of ABD will be needed in the future. In the event of ethical concerns, further surveys in conjunction with figures on the frequency of certain diseases and complications following dental procedures may provide a possible decision‐making aid. In addition, the dissemination of guidelines among (dental) medical personnel should be further promoted to ensure compliance.

## Author Contributions

The study was developed by Joshua Kinzel and Roland Seifert together. Data collection and analysis were carried out by Joshua Kinzel. The manuscript was written by Joshua Kinzel and commented on and approved by Roland Seifert.

## Funding

No funding was received for this project. Open Access funding enabled and organized by Projekt DEAL.

## Disclosure

The authors declare that all data were generated internally.

## Ethics Statement

This study was conducted in accordance with the guidelines of good scientific practice of the Hannover Medical School (https://www.mhh.de/forschung/gute-wissenschaftliche-praxis) and officially registered as part of a doctoral project for Joshua Kinzel. The Hannover Medical School follows the guidelines of the German Research Foundation (DFG).

## Consent

The authors have nothing to report.

## Conflicts of Interest

The authors declare no conflicts of interest.

## Supporting Information

Additional supporting information can be found online in the Supporting Information section.

## Supporting information


**Supporting Information** A survey of German dentists on the prophylactic use of antibacterial drugs in patients in risk groups: diabetes, joint replacement, risk of endocarditis and immunosuppression or organ transplantation.

## Data Availability

All source data for this study are available upon reasonable request.
