# Supplementary material for: A Survey of German Dentists on the Prophylactic Use of Antibacterial Drugs in Patients in Risk Groups: Diabetes, Joint Replacement, Risk of Endocarditis, and Immunosuppression or Organ Transplantation
Source: Int J Dent. 2026 Jun 19;2026:8452465. doi: 10.1155/ijod/8452465 (PMC13280459; doi:10.1155/ijod/8452465)
Supplement: Supplementary file 1 — Supporting Information A survey of German dentists on the prophylactic use of antibacterial drugs in patients in risk groups: diabetes, joint replacement, risk of endocarditis and immunosuppression or organ transplantation. [file IJOD-2026-8452465-s001.docx]

**Supplement**

**A survey of German dentists on the prophylactic use of antibacterial drugs in patients in risk groups: diabetes, joint replacement, risk of endocarditis and immunosuppression or organ transplantation**

**Joshua Kinzel and Roland Seifert**

Info AntibiotikaprophylaxeZahnaerzte Statistische Erfassung prophylaktisch genutzter Antibiotika bei Zahnärzten

Info base Fragebogen

Page 1 Seite 01 Seite 1

Qst I001 Instruktionstext

Ttl Mit dieser Umfrage soll der Umgang mit prophylaktisch verordneten Antibiotika im realen Praxisalltag statistisch erfasst werden. Die Umfrage dauert ca. 5-10 Minuten. Mit Ihrer Teilnahme leisten Sie einen wichtigen Beitrag zu meiner Forschung. Ich freue mich daher sehr, wenn Sie an meiner Umfrage teilnehmen. Die Teilnahme ist freiwillig. Alle Angaben die Sie machen sind anonym und werden nicht auf Einzelfallebene ausgewertet.

Val 1=nicht gewählt, 2=ausgewählt

Itm I001_01 Ich stimme der Teilnahme zu.

Page Seite 02

Qst G001 Antibiotika bei Gelenkersatz

Ttl Nutzen Sie Antibiotika zur prophylaktischen Abschirmung bei Patienten mit Gelenkersatz?

Val "1=nie (0%), 2=selten (&gt;25%), 3=gelegentlich (25-50%), 4=häufig (50-75%), 5=sehr oft (&lt;75%), 6=immer (100%), 7=keine Angabe, ..."

Itm G001_01 Bei zahnärztlichen Eingriffen innerhalb von zwei Jahre nach Gelenkersatzoperation

Itm G001_02 Über zwei Jahre nach Gelenkersatzoperation hinaus

Itm G001_03 Bei Hüftgelenkersatz

Itm G001_04 Bei Patienten mit Gelenkersatz in Verbindung mit weiteren Risikofaktoren (Rauchen, starker Alkoholkonsum)

Itm G001_05 Bei Zahnentfernung

Itm G001_06 Bei Implantation

Itm G001_07 Bei Entzündungen des Endodonts

Itm G001_08 Bei Entzündungen der Mundschleimhaut

Itm G001_09 Bei parodontalen Eingriffen

Itm G001_10 Bei Zahn- oder Kiefertraumata

Itm G001_11 Bei Verletzung der Mukosa

Itm G001_12 Bei Verletzung der Gingiva

Itm G001_13 Bei lokalanästhetischer Injektion

Itm G001_14 Bei Sondierung des PA-Spalts

Itm G001_15 Bei postoperativer Nahtentfernung

Itm G001_16 Bei Eingriffen mit Knochenbeteiligung

Page Seite 03

Qst GA01 Antibiotikum bei Gelenkersatz

Ttl Welches Antibiotikum nutzen Sie zur prophylaktischen Abschirmung von Patienten mit Gelenkersatz am häufigsten?

Itm GA01_01 Amoxicillin

Itm GA01_02 Clindamycin

Itm GA01_03 Penicillin V

Itm GA01_04 Doxycyclin

Itm GA01_05 Metronidazol

Itm GA01_06 Ampicillin

Itm GA01_07 Vancomycin

Itm GA01_08 Azithromycin

Itm GA01_09 Clarithromycin

Page Seite 04

Qst E001 Antibiotika zur Endokarditisprophylaxe

Ttl Nutzen Sie Antibiotika zur Endokarditisprophylaxe?

Val "1=nie (0%), 2=selten (&lt;25%), 3=gelegentlich (25-50%), 4=häufig (50-75%), 5=sehr oft (&gt;75%), 6=immer (100%), 7=keine Angabe, ..."

Itm E001_01 Bei Patienten zyanotischem Herzfehler

Itm E001_02 Bei Patienten mit prothetischer Herzklappe

Itm E001_03 Bei Patienten mit überstandener bakterieller Endokarditis

Itm E001_04 Bei herztransplantierten Patienten

Itm E001_05 Bei zyanotischen angeborenen Herzkrankheiten

Itm E001_06 Bei chirurgisch angelegten pulmonalen Shunts

Itm E001_07 Bei anderen angeborenen Herzfehlern (außer den Zyanotischen)

Itm E001_08 Bei erworbener Valvar-Dysfunktion (z.B. rheumatische Herzkrankheit)

Itm E001_09 Hypertrophe Kardiomyopathie

Itm E001_10 Bei Mitralklappenprolaps

Itm E001_14 Bei einer, in der Umfrage nicht aufgeführten Herzerkrankung

Itm E001_11 Bei einer oben genannten Erkrankung in Verbindung mit weiteren Risikofaktoren (Rauchen, starker Alkoholkonsum)

Itm E001_12 Bei Zahnentfernung

Itm E001_13 Bei Implantation

Itm E001_15 Bei Entzündungen des Endodonts

Itm E001_16 Bei Entzündungen der Mundschleimhaut

Itm E001_17 Bei parodontalen Eingriffen

Itm E001_18 Bei Zahn- oder Kiefertraumata

Itm E001_19 Bei Verletzung der Mukosa

Itm E001_20 Bei Verletzung der Gingiva

Itm E001_21 Bei lokalanästhetischer Injektion

Itm E001_22 Bei Sondierung des PA-Spalts

Itm E001_23 Bei postoperativer Nahtentfernung

Itm E001_24 Bei Eingriffen mit Knochenbeteiligung

Page Seite 05

Qst EA01 Antibiotikum zur Endokarditisprophylaxe

Ttl Welches Antibiotikum nutzen Sie zur Endokarditisprophylaxe am häufigsten?

Itm EA01_01 Amoxicillin

Itm EA01_02 Clindamycin

Itm EA01_03 Penicillin V

Itm EA01_04 Doxycyclin

Itm EA01_05 Metronidazol

Itm EA01_06 Ampicillin

Itm EA01_07 Vancomycin

Itm EA01_08 Azithromycin

Itm EA01_09 Clarithromycin

Page Seite 06

Qst D001 Antibiotika bei Diabetes

Ttl Nutzen Sie Antibiotika zur Prophylaxe bei Patienten mit Diabetes?

Val "1=nie (0%), 2=selten (&lt;25%), 3=gelegentlich (25-50%), 4=häufig (50-75%), 5=sehr oft (&gt;75%), 6=immer (100%), 7=keine Angabe, ..."

Itm D001_01 Bei Diabetes mellitus Typ 1

Itm D001_02 Bei Diabetes mellitus Typ 2

Itm D001_03 Bei schlecht eingestelltem Diabetes

Itm D001_04 Bei Diabetes in Verbindung mit weiteren Risikofaktoren (Rauchen, starker Alkoholkonsum)

Itm D001_05 Bei Zahnentfernung

Itm D001_06 Bei Implantation

Itm D001_07 Bei Entzündungen des Endodonts

Itm D001_08 Bei Entzündungen der Mundschleimhaut

Itm D001_09 Bei parodontalen Eingriffen

Itm D001_10 Bei Zahn- oder Kiefertraumata

Itm D001_11 Bei Verletzung der Mukosa

Itm D001_12 Bei Verletzung der Gingiva

Itm D001_13 Bei lokalanästhetischer Injektion

Itm D001_14 Bei Sondierung des PA-Spalts

Itm D001_15 Bei postoperativer Nahtentfernung

Itm D001_16 Bei Eingriffen mit Knochenbeteiligung

Page Seite 07

Qst DA01 Antibiotikum bei Diabetes

Ttl Welches Antibiotikum nutzen Sie zur prophylaktischen Abschirmung von Patienten mit Gelenkersatz am häufigsten?

Itm DA01_01 Amoxicillin

Itm DA01_02 Clindamycin

Itm DA01_03 Penicillin V

Itm DA01_04 Doxycyclin

Itm DA01_05 Metronidazol

Itm DA01_06 Ampicillin

Itm DA01_07 Vancomycin

Itm DA01_08 Azithromycin

Itm DA01_09 Clarithromycin

Page Seite 08

Qst O001 Antibiotika bei Immunsuppression oder Organtransplantation

Ttl Nutzen Sie Antibiotika zur Prophylaxe bei Immunsuppression oder nach Organtransplantation?

Val "1=nie (0%), 2=selten (&lt;25%), 3=gelegentlich (25-50%), 4=häufig (50-75%), 5=sehr oft (&gt;75%), 6=immer (100%), 7=keine Angabe, ..."

Itm O001_01 Bei Patienten mit immunsupprimierender Erkrankung

Itm O001_02 Bei Patienten mit immunsupprimierender Medikation

Itm O001_03 Bei Patienten nach Organtransplantation

Itm O001_04 Bei Immunsuppression oder nach Organtransplantation in Verbindung mit weiteren Risikofaktoren (Rauchen, starker Alkoholkonsum)

Itm O001_05 Bei Zahnentfernung

Itm O001_06 Bei Implantation

Itm O001_07 Bei Entzündungen des Endodonts

Itm O001_08 Bei Entzündungen der Mundschleimhaut

Itm O001_09 Bei parodontalen Eingriffen

Itm O001_10 Bei Zahn- oder Kiefertraumata

Itm O001_11 Bei Verletzung der Mukosa

Itm O001_12 Bei Verletzung der Gingiva

Itm O001_13 Bei lokalanästhetischer Injektion

Itm O001_14 Bei Sondierung des PA-Spalts

Itm O001_15 Bei postoperativer Nahtentfernung

Itm O001_16 Bei Eingriffen mit Knochenbeteiligung

Page Seite 09

Qst OA01 Antibiotikum bei Immunsupression oder Organtransplantation

Ttl Welches Antibiotikum nutzen Sie zur prophylaktischen Abschirmung von Patienten mit Gelenkersatz am häufigsten?

Itm OA01_01 Amoxicillin

Itm OA01_02 Clindamycin

Itm OA01_03 Penicillin V

Itm OA01_04 Doxycyclin

Itm OA01_05 Metronidazol

Itm OA01_06 Ampicillin

Itm OA01_07 Vancomycin

Itm OA01_08 Azithromycin

Itm OA01_09 Clarithromycin

Page Seite 10

Qst S001 Sicherheit im Umgang

Ttl Fühlen Sie sich sicher im Umgang mit der Anwendung von Antibiotika zu Prophylaxezwecken?

Itm S001_01 ja

Itm S001_02 nein

Page Seite 11

Qst EX01 Jahr des Staatsexamens

Ttl In welchem Jahr haben Sie Ihr Staatsexamen Zahnmedizin abgelegt?

Itm EX01_01 2023

Itm EX01_02 2022

Itm EX01_03 2021

Itm EX01_04 2020

Itm EX01_05 2019

Itm EX01_06 2018

Itm EX01_07 2017

Itm EX01_08 2016

Itm EX01_09 2016

Itm EX01_10 2015

Itm EX01_11 2014

Itm EX01_12 2013

Itm EX01_13 2012

Itm EX01_14 2011

Itm EX01_15 2010

Itm EX01_16 2009

Itm EX01_17 2008

Itm EX01_18 2007

Itm EX01_19 2006

Itm EX01_20 2005

Itm EX01_21 2004

Itm EX01_22 2003

Itm EX01_23 2002

Itm EX01_24 2001

Itm EX01_25 2000

Itm EX01_26 1999

Itm EX01_27 1998

Itm EX01_28 1997

Itm EX01_29 1996

Itm EX01_30 1995

Itm EX01_31 1994

Itm EX01_32 1993

Itm EX01_33 1992

Itm EX01_34 1991

Itm EX01_35 1990

Itm EX01_36 1989

Itm EX01_37 1988

Itm EX01_38 1987

Itm EX01_39 1986

Itm EX01_40 1985

Itm EX01_41 1984

Itm EX01_42 1983

Itm EX01_43 1982

Itm EX01_44 1981

Itm EX01_45 1980

Itm EX01_46 1979

Itm EX01_47 1978

Itm EX01_48 1977

Itm EX01_49 1976

Itm EX01_50 1975

Itm EX01_51 1974

Itm EX01_52 1973

Itm EX01_53 1972

Itm EX01_54 1971

Itm EX01_55 1970

Itm EX01_56 1969

Itm EX01_57 1968

Itm EX01_58 1967

Itm EX01_59 1966

Itm EX01_60 1965

Itm EX01_61 1964

Itm EX01_62 1963

Itm EX01_63 1962

Itm EX01_64 1961

Itm EX01_65 1960

Itm EX01_66 1959

Itm EX01_67 1958

Itm EX01_68 1957

Itm EX01_69 1956

Itm EX01_70 1955

Itm EX01_71 1954

Itm EX01_72 1953

Itm EX01_73 1952

Itm EX01_74 1951

Itm EX01_75 1950
